# Self-Management of Diabetes and Associated Factors among Patients Seeking Chronic Care in Tshwane, South Africa: A Facility-Based Study

**DOI:** 10.3390/ijerph20105887

**Published:** 2023-05-19

**Authors:** Janke Zwane, Perpetua Modjadji, Sphiwe Madiba, Lucky Moropeng, Kabelo Mokgalaboni, Peter Modupi Mphekgwana, Andre Pascal Kengne, Zandile June-Rose Mchiza

**Affiliations:** 1 Department of Public Health, School of Health Care Sciences, Sefako Makgatho Health Sciences University, 1 Molotlegi Street, Ga-Rankuwa 0208, South Africa; 2 Non-Communicable Disease Research Unit, South African Medical Research Council, Tygerberg 7505, South Africa; 3 Faculty of Health Sciences, University of Limpopo, Polokwane 0700, South Africa; 4 Faculty of Health Sciences, School of Health Systems and Public Health Care Sciences, University of Pretoria, 31 Bophelo Road, Gezina 0031, South Africa; 5 Department of Life and Consumer Sciences, College of Agriculture and Environmental Sciences, University of South Africa, Florida 1710, South Africa; 6 Research Administration and Development, University of Limpopo, Polokwane 0700, South Africa

**Keywords:** diabetes, self-management, associated factors, primary health care facilities, South Africa

## Abstract

The burden of diabetes continues to increase in South Africa and a significant number of diabetes patients present at public primary healthcare facilities with uncontrolled glucose. We conducted a facility-based cross-sectional study to determine the diabetes self-management practices and associated factors among out-patients in Tshwane, South Africa. An adapted validated questionnaire was used to collect data on sociodemography, diabetes knowledge, and summaries of diabetes self-management activities measured in the previous seven days, and over the last eight weeks. Data were analysed using STATA 17. A final sample of 402 diabetes out-patients was obtained (mean age: 43 ± 12 years) and over half of them were living in poor households. The mean total diabetes self-management of score was 41.5 ± 8.2, with a range of 21 to 71. Almost two thirds of patients had average self-management of diabetes, and 55% had average diabetes knowledge. Twenty-two percent of patients had uncontrolled glucose, hypertension (24%) was the common comorbidity, and diabetic neuropathy (22%) was the most common complication. Sex [male: AOR = 0.55, 95% CI: 0.34–0.90], race [Coloured: AOR = 2.84, 95% CI: 1.69–4.77 and White: AOR = 3.84, 95% CI: 1.46–10.1], marital status [divorced: AOR = 3.41, 95% CI: 1.13–10.29], social support [average: AOR = 2.51, 95% CI: 1.05–6.00 and good: AOR = 4.49, 95% CI: 1.61–7.57], body mass index [obesity: AOR = 0.31, 95% CI: 0.10–0.95], diabetes knowledge [average: AOR = 0.58, 95% CI: 0.33–0.10 and good: AOR = 1.86, 95% CI: 0.71–4.91], and uncontrolled glucose [AOR = 2.97, 95% CI: 1.47–5.98] were factors independently predictive of diabetes self-management. This study emphasizes that the self-management of diabetes was mostly on average among patients and was associated with the aforementioned factors. Innovative approaches are perhaps needed to make diabetes education more effective. Face-to-face sessions delivered generally during clinic visits should be better tailored to the individual circumstances of diabetes patients. Considerations should be given to the options of leveraging information technology to ensure the continuity of diabetes education beyond clinic visits. Additional effort is also needed to meet the self-care needs of all patients.

## 1. Introduction

Type 2 diabetes (T2DM) is one of the major global issues currently affecting 415 million people, and is projected to rise to 642 million by 2040 [1]. Over 90% of T2DM cases are observed in low and middle-income countries (LMICs) [2,3,4]. The greatest increase of T2DM can be observed in Africa, particularly in the sub-Saharan Africa (SSA) region, with 14.2 million cases (20–79 years). This number is expected to rise to 34.2 million by 2040 [1,5]. This progression is attributed to the epidemiological transition due to the adoption of the western lifestyle and urbanization, among other influences [5]. In addition, SSA has the highest proportion of undiagnosed cases of T2DM and over two-thirds of people affected are not aware of their status [1]. Proper management of diabetes is imperative, yet, SSA is compounded by challenges of poor management, limited resources, coexisting traditional health priorities, and low health insurance coverage [6] contributing to complications such as nephropathy, retinopathy, and peripheral neuropathy [7]. These complications are mostly linked to diabetic foot disease, eventually leading to amputation (i.e., microvascular changes) [7], as well as cardiovascular disease, cerebrovascular disease, strokes, and coronary heart disease (macrovascular injuries) [8]. Therefore, the growing burden of T2DM remains a barrier to the wellbeing of affected individuals and families, and to the proper functioning of the health system [2,4].

To date, South Africa has experienced alarming rates of T2DM, which have tripled from 4.5% in 2010 to 12.7% in 2019, and are currently estimated at 4.58 million people (aged 20 and 79 years) living with T2DM, of which 52.4% are undiagnosed [9], and 65% of T2DM cases [10]. In 2014, the Centralised Chronic Medicine Dispensing and Distribution Programme (CCMDD) was launched by the South African National Department of Health to improve access to medication and patient adherence [11]. However, gaps in the T2DM management programme showed that only 29% of patients had acceptable blood glucose levels [11]. Furthermore, effective as of 1 April 2017, the South African government has implemented a sugar tax, subjecting sugar-sweetened beverages to a tax based on their sugar content [12]. The aim of sugar tax was to curb the over consumption of sugar in the population, which has been linked to the growing burden of non-communicable diseases (NCDs), especially T2DM [12].

The literature documents that self-management, including lifestyle changes among patients with T2DM, may be a more efficient treatment strategy for patients due to a strong correlation between T2DM and unhealthy lifestyle, and may also assist health care providers in their duties [13,14,15]. Self-management is defined as the active participation of patients in their treatment, with three distinct sets of self-management activities: taking medication and adhering to dietary advice, adopting new behaviours in the context of a chronic disease, and dealing with the feelings of frustration, fright, and despair associated with chronic disease [16,17]. However, self-management support remains relatively under-developed in most countries [18], despite optimal support being essential in chronic care [19]. For instance, one of the main goals of T2DM management is to achieve glycaemic control to delay or prevent the onset of diabetes complications [20]. Thus far, South Africa has recorded poor glycaemic control, ranging from 7.6% to 83.6% [21,22,23], with a current prevalence at 77.7% [24], while in SSA, poor glycaemic control among T2DM patients has been estimated at an average of 60% [20]. This is attributed to low awareness of the disease, sub-optimal treatment, and fragmented health systems [25,26]. Countries such as Spain (45%), Belgium (54%), Germany (61%), and the United States of America (55%) have observed good glycaemic control [27,28], which is attributed to access to resources and support systems, patients’ attitude and expected treatment efforts, comorbidities, disease duration, and risk of hypoglycaemia [29].

It is worth noting that the strategic plans and policies for long-term care and self-management in South Africa are predominated by the self-responsibility of chronic disease patients, and adequate support from primary health care teams to teach and empower patients for appropriate disease self-management [30]. However, the country continues to experience a high number of patients presenting at primary health care (PHC) facilities with poorly controlled T2DM [31] seeking chronic care for related complications mostly entailing lower limb amputations and loss of sight, yet researchers have proven beyond doubt that normal glycaemic control is crucial to prevent diabetes consequences in South Africa [24,32,33]. These consequences have substantial effects on quality of life regarding the increasing medical and rehabilitative costs that come with hospitalisation, medication, and outpatient visits, combined with a loss of income due to the inability to work, among other issues [34,35,36]. There is a need to engage preventive measures to alleviate the burden of diabetes in South Africa and emphasise optimum screening for prediabetes in line with the South African guidelines [37]. The South African National Department of Health [37] recommends screening for pre-diabetes as a way to prevent diabetes, which is best addressed by lifestyle modification [38], in addition to using proper medications, low calorie intake of bad foods such as via the consumption of a low salt diet, as well as physical activity and reduction in body weight.

Nonetheless, poor self-management of diabetes continues to be a major public health concern in LMICs, SSA, and South Africa, implicating different contexts and factors [20,24,34,39,40,41,42]. This makes continuous, translational, and contextual research imperative to determine the level of compliance to self-management activities and the associated factors among diabetes patients necessary to highlight a need to enhance the existing plan of action in addressing diabetes and its consequences. Contextual risk factors impact health and consider person, place, and time, including social and environmental risk factors beyond the individual level, especially sociodemographic factors [43]. Additionally, it is important to bear in mind that challenges to the management of diabetes revolve around optimal care, diabetes self-management education, medication adherence, reducing barriers, and improving health delivery [44]. There has been minimal research conducted on contextual factors regarding diabetes self-management in the country, particularly the practice of foot-care, which is vital to prevent limb amputation; one of the most common complications (71.8%) of diabetes in South Africa [45].

Additionally, emphasis has been placed on the qualitative aspects of diabetes self-management, instead of offering quantitative data. Lastly, this study investigated the self-management of diabetes at public PHC facilities; the first line of treatment for many South Africans, and not in the tertiary hospitals that have been well-researched in the past. According to the South African Statista Research Department [46], as of 2021, 16.1% of South Africans were members of medical aid schemes, and white individuals (77.7%) and Indians/Asians (45.1%) were mostly covered compared to the Coloureds (19.9%) and Black Africans (9.3%). Hence, most South Africans use public PHC facilities for programmes such as CCMDD to access treatment for chronic care [11]. In this regard, we conducted a facility-based cross-sectional study to determine the diabetes self-management practices and associated factors among out-patients in Tshwane, South Africa.

## 2. Materials and Methods

### 2.1. Study Design and Framework

A cross-sectional study was conducted among diabetes out-patients seeking chronic care at Tshwane public PHC facilities in South Africa. The study was conducted between September 2021 and March 2022. The study was anchored on the conceptual frameworks of both Brown et al. [47] and Sousa et al. [48] to investigate the link between socioeconomic and other factors related to diabetes mellitus, diabetes knowledge, diabetes self-management practices, and health outcomes, which informed data collection and analysis. Brown et al. [47] mentioned socioeconomic factors such as education level and employment status in their framework, together with critical covariates such as age and sex, which are mediated by factors such as self-management practices and health behaviours. These health behaviours entail diet adherence, access to health care, and processes of foot care, leading to certain health outcomes and complications. On the other hand, Sousa et al. [48] simplified the framework and showed a linear relationship between diabetes knowledge, social support, self-management agency (self-management capability), and self-efficacy (a diabetes sufferer’s belief in his/her own self-management capability). These two frameworks summarise the relationship between diabetes components with several demographic and lifestyle factors, and informed data collection and analysis in this study.

### 2.2. Study Setting and Population

This study was conducted at the City of Tshwane (CoT) in the northern Gauteng Province, South Africa [49]. CoT is surrounded by towns and localities included in the local government area and the area has 22 public PHC facilities [49,50]. According to Statistics South Africa [51], Africans make up the greatest proportion of 75.40%, followed by White (20.08%), then Coloured (2.01%), and Indian or Asian (1.84%), living in the informal settlements, peri-urban, urban, and rural settings of CoT. The study included out-patients who were 18 years and above, who were able to give consent, were diagnosed with diabetes, and were on medication for a year or more prior to data collection, and were receiving out-patient care from one of the identified PHC facilities. Out-patients who did not meet the aforementioned criteria were excluded from the study.

### 2.3. Sample Size and Sampling Techniques

Anecdotal information from the respective facilities estimates diabetes patients at 269,700 patients. The population seeking health care at these facilities is often of a lower to middle socioeconomic class and many present with poor to moderate literacy levels. Considering a confidence level of 95%, Cochran’s formula determined a minimum sample of 384 participants. A final sample size of 402 patients was eventually obtained after excluding eight questionnaires with more than 10% missing data. Cluster sampling was used to select PHC facilities in CoT and this allowed for widespread representative inclusion of out-patients to represent different areas. Ultimately, four public PHC facilities were used in this study (Mamelodi, Eersterust, Saulsville, and Skinner). PHC facilities offer first line treatment for patients suffering from chronic diseases diagnosed by medical doctors and nursing staff [35], nonetheless, these facilities continue to experience overcrowding, long waiting times, and a lack of resources, such as medicine stock-outs [37]. In 1994, the South African Ministry of Health adopted the PHC approach to health care delivery as a fundamental system to advance health care to the population through accessible services and effective use [52,53].

**Figure 1:**
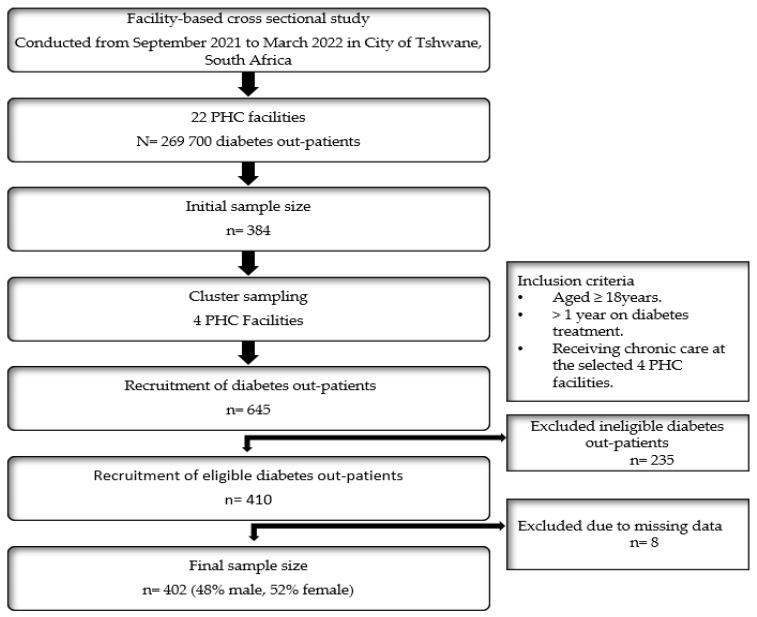
Study design, recruitment, and sampling processes.

While sampling out-patients waiting in queues for services in the facilities, the difficulty of obtaining a random sample became evident. This led us to using convenience sampling to select patients who met the inclusion criteria. Although convenience sampling is a non-probability sampling method, it is the most applicable and widely used method in clinical research [54]. After obtaining the permission to conduct the study from the health facilities, the main researcher recruited and engaged out-patients on the procedure and preparations for data collection with the help of the facility manager and two research assistants. After diabetes patients received sufficient information about the study and voluntarily provided written consent to participate in the study, data collection commenced in the PHC facilities without interrupting the daily health services. The recruitment and sampling processes were performed for PHC facilities and out-patients without considering any facility as a unit of analysis (Figure 1).

### 2.4. Data Collection and Tool

#### 2.4.1. Sociodemographic Data and Diabetes Measures, Activities, and Knowledge

A self-administered questionnaire (see Supplementary Materials) was distributed by the main researcher and the research assistants to out-patients for completion. The first section of the self-administered questionnaire contained various general and socioeconomic questions pertaining to topics such as age, sex, race, and household income. This enabled us to determine the sociodemographic factors associated with self-management practices in diabetes patients. Secondly, the diabetic knowledge of the patients was assessed. Thirdly, self-management practices and compliance were evaluated. Certain sections regarding the three existing diabetes self-management questionnaires were adapted with consideration that the questionnaire has previously been tested for reproducibility and repeatability. The fourth section considered anthropometry and physiological measurements. Quality assessment was performed using the 22-items “Strengthening the Reporting of Observational Studies in Epidemiology” (STROBE) checklist [55]. Independent translators who speak isiZulu as their mother tongue and are conversant with English performed forward and backward translations of the questionnaire, while the main researcher, whose first language is Afrikaans, translated the questionnaire from English to Afrikaans and back. Some of the questions in these tools may have been rather challenging for people of a lower educational or literacy level to fully understand. Therefore, the main researcher and research assistants were available to clarify or assist patients with answering the questionnaire, should they have had further queries and/or been unable to understand the questions due to poor literacy levels, poor eyesight, or any language barriers.

A pilot study [56] was conducted among a few outpatients to pre-test the questionnaire and determine its feasibility in one of the facilities that did not form part of the study, and the results were not included in the data analysis for the main study. Prior to the pilot study, the research assistants who speak isiZulu, English, and Afrikaans were taken through the process of conducting preliminary interviews to assist the patients if need be. During the pilot study, research assistants were trained to take physical measurements by the main researcher, who also assisted with Afrikaans-speaking patients.

A questionnaire was adapted from the revised brief Diabetes Knowledge Test (DKT2), summary of Diabetes Self-Management Activities Measure (SDSCA) [57], and Diabetes Self-Management Questionnaire (DSMQ) [31]. Ten questions from the Diabetes Knowledge Test (DKT2) were selected to briefly test the knowledge of the diabetic participants before investigating their self-management practices using SDSCA and DSMQ tools. Several questions from each of these tools were selected and combined to create a new data-collection tool for this study. No single section was solely used, since it would be limited to determining the self-management practices of diabetes and the associated factors in South Africa. Scores of diabetes knowledge among the patients were sorted into three categories: poor (0–3 scores), average (4–6 scores), and good (7–10 scores). Self-management activities over the past seven days (using SDSCA) prior to data collection were assessed based on the diet, exercise, blood sugar testing, medication, foot care, smoking, and alcohol consumption of the participants. Furthermore, self-management activities related to diabetes were assessed over the last eight weeks (using DSMQ). Combined scores of self-management activities and compliance were assessed using a Likert scale to rate the extent to which patients agreed to certain statements pertaining to their diabetes self-management activities from “applies to me very much” to “does not apply to me” to compute the self-management variable. This variable considered total diet score (=17), total exercise score (=17), total medication score (=12), total testing score (=12), footcare (=7), smoking (=1), alcohol (=1), doctor (=5), and self-care (=5), giving a total self-management score = 77. Assessed self-management was categorised as poor (0–37), fair (38–57), and good (58–77).

#### 2.4.2. Anthropometric and Physiological Measurements

Weight was measured using a well-calibrated electronic scale (Elektra DLK Sports Electronic Body Stat Scale) and height was measured using a height measuring board, and both were rounded to one decimal point. Body mass index (BMI) was calculated from weight in kilograms (kg) divided by the height in meters squared (m^2^). Underweight was BMI below 18.9 kg/m^2^, normal weight was BMI of 19–24.9 kg/m^2^, overweight was BMI of 25–29.9 kg/m^2^, and obesity was BMI of more than or equal to 30 kg/m^2^ [58]. Waist (WC) and hip (HC) circumferences were measured using a non-stretchable measuring tape, rounded to one decimal, and recorded in centimeters. WC and HC were used to calculate waist-hip ratio (WHR), while waist-to-height ratio (WHtR) was computed. All measurements were taken three times, according to the World Health Organization (WHO) recommendations, and the average values were recorded. Abdominal obesity was assessed using the following: WC [Females: <88 (normal) and Males: <94 cm, and Females: ≥88, Males: ≥94 cm (abdominal obesity)], WHR [females: <0.85 (normal) and males: 0.94 (normal), and females: ≥0.85, males ≥ 0.94 (abdominal obesity)], and WHtR [<0.5 (normal) ≥0.5, and (abdominal obesity)] [58,59].

Physiological measurements of blood glucose levels and blood pressure were also taken, unless these measurements could be found recorded in the medical file of the participant, especially taken on the same day of data collection. Capillary blood was taken with a finger prick using a disposable lancet and measured twice using a well-calibrated electronic glucose meter (Fora Diamond model), and the average glucose level was considered. A blood glucose level ≥7 mmol/L was considered as uncontrolled diabetes [60]. Blood pressure was measured using a sphygmomanometer (manufactured by Braun) and recorded as systolic (SBP) over diastolic (DBP) blood pressure measurements, using the unit of millimeters of mercury (mmHg). A blood pressure level of ≥140/90 mmHg was considered as hypertension [61], a risk factor for diabetes and complications [62].

### 2.5. Data Analysis

Data were analysed using STATA version 17 (StataCorp. 2021. Stata Statistical Software: Release 17., StataCorp LLC, College Station, TX, USA). Missing data were identified through complete case analysis. Descriptive statistics was performed to generate the frequencies (*n*) and proportions (%) of sociodemographic data, medical status, and diabetes knowledge. Diabetes knowledge scores among the participants were grouped into three categories: poor (0–3 scores), average (4–6 scores), and good (7–10 scores), as well as mean ± SD. Assessed self-care management activities are presented as means ± SD, frequencies, and proportions, and categorised as poor (0–3 scores), fair (4–5 scores), and good (6–7 scores), while exercise on five or more days was considered good. Combined scores were used to assess self-management practices and compliance, considering total diet score, total exercise score, total medication score, total testing score, total testing score, and total self-care score, and the total calculated self-management variable was categorized into three groups: 0 to 37 (poor), 38 to 57 scores (fair), and 58 to 77 (good). The inversion scores of the number of days high fat products were eaten was used, and a greater number of days suggested a poor diet. Chi square and Fisher’s exact tests were used to determine the association of self-management with selected covariates (such as sociodemographic factors, medical status, and anthropometry). The results are presented as *n* (%) and a probability level of 0.05 indicated significance. For logistic regression analysis, dichotomized average self-management, which is an outcome variable, was created through combining good and fair self-management categories to form one category, while poor self-management was a second category on its own. The *p*-value was statistically significant at less than 0.05. Results are presented as frequencies (*n*) and percentages (%), crude odds ratio [OR (95%CI)], and adjusted odds ratio [AOR; (95%CI)].

### 2.6. Ethical Considerations

This study was conducted according to the guidelines laid down in the Declaration of Helsinki [63] and all procedures involving human subjects were approved by Sefako Makgatho Health Sciences University Research and Ethics Committee (SMUREC); (SMUREC/H/62/2021: PG) on 6 May 2021. Furthermore, this study received permission from the Tshwane Research Committee (Reference number: GP_2021_024). Written informed consent was obtained from all the patients prior to data collection.

## 3. Results

### 3.1. Characteristics of Out-Patients and Comparison by Sex

Four hundred and two (*n* = 402) out-patients diagnosed with diabetes; 210 (52%) females and 192 (48%) males participated in the study. The mean age of the patients was 43 ± 12 years, and 252 (63%) of them were aged 45 years and below, while 147 (37%) were above 45 years. Most patients were living in urban (72%) compared to peri-urban areas (28%). Most patients were black Africans (45%), followed by Coloureds (40%), and thirty-five percent (35%) of the patients were single, and 44% were married. Secondary school level (34%) and grade 12 level (48%) were the most frequently attained education levels. Most patients were living in households with monthly income between 83.11–276.85 $ (53%), while 70% reported good social support from family and friends. Forty nine percent (49%) of the patients indicated that they were diagnosed with diabetes with the past five years, whilst 51% were diagnosed for more than five years. About 8% of the patients had comorbid conditions, and hypertension and cholesterol were the most common coexisting conditions with diabetes. Common comorbidities among the patients were hypertension (24%) and high cholesterol (21%). The most prevalent complications were neuropathy (22%) and loss of eyesight (16%), while 23% of the patients reported to have been hospitalised at some point in time. The most common medication was oral hypoglycaemic tablets (49%), followed by insulin (28%), and combined oral hypoglycaemic tablets and insulin (21%). Half of the patients had access to a blood glucose machine to self-monitor at home. Over half of the patients [*n* = 222 (55%)] had an average diabetes knowledge. Significant differences of patients’ characteristics by sex were observed for marital status (*p* = 0.005), neuropathy complication (*p* = 0.029), ever hospitalised (*p* = 0.043), use oral hypoglycaemics tablets (*p* ≤ 0.0001), and insulin (*p* ≤ 0.0001) medication (Table 1).

### 3.2. Glucose, Blood Pressure and Anthropometric Measurements of Patients

Table 2 shows a comparison of the anthropometric and physiological measurements of patients by sex, within, under, or above the cut-off points; Glucose [<7 mmol/L (controlled) and ≥7 mmol/L (uncontrolled)], Hypertension [SBP/DBP: 120/80 (normal) and ≥140/90: (hypertensive)], BMI [18.5–24.9 (normal), 25–29.9 (overweight) ≥30 (obese)], WC [females: <88 (normal) and males: <94 cm, and females: ≥88, males: ≥94 cm (abdominal obesity)], WHR [females: <0.85 (normal) and males: 0.94 (normal), and females: ≥0.85, males: ≥0.94 (abdominal obesity)], and WHtR [<0.5 (normal) ≥0.5, and (abdominal obesity)]. Uncontrolled glucose was significantly higher in the older patients (37%) compared to the other age group (13%), *p*≤0.001 (results not shown in table). Twenty-two percent of the patients (22%) had uncontrolled glucose levels, and females (27%) were significantly affected compared to males (17%), *p* = 0.012. The prevalence of hypertension was 29%, while overweight (30%) and obesity (63%) were high among the patients, with females (70%) being significantly obese versus males (55%), *p* = 0.007, and no one was under weight.

### 3.3. Self-Care Management Activities of the Patients

As displayed in Table 3, the self-management activities over the past seven days relative to the time of data collection were assessed based on diet, exercise, blood sugar self-testing, medication, foot care, smoking, and alcohol consumption (using SDSCA). The results are presented as mean ± SD, and frequencies and proportions [*n* (%)], and categorised as poor (0–3 scores), fair (4–5 scores), and good (6–7 scores), and exercise on five or more days was categorized as good. Women who drink >1 drink/day were regarded as heavy drinkers, and those who drink <1 drink/day was regarded as light drinkers. Men who drink >2 drinks/day were categorized as heavy drinkers, and men who drink <2 drinks/day were light drinkers. Forty-seven percent of the patients had poor consumption of fruit and vegetables in the past seven days, while 49% consumed high fat foods, and 70% did not exercise. Furthermore, blood glucose self-testing (93%), foot care (60%), and shoe checks (70%) among the patients were poor in the past seven days. At least 67% of the patients adhered to medication in the seven days, although 33% of the patients reported heavy drinking, while 25% reported heavy smoking.

In Table 4, the combined scores of self-management activities and compliance were assessed over the last eight weeks using DSMQ. The assessed self-care management activities are presented as means ± SD, frequencies, and proportions, and categorised as poor (0–37), fair (38–57), and good (58–77). The results showed that only 10% had a good total diet, 10% were good with exercise, 75% had a good total medication score, and only 3% had a good total self-testing score. A total of 31% had a poor self-management score, with a mean of 41.5 ± 8.2, ranging from 21 to 71 scores, but most were average, among 64% of the patients.

### 3.4. Factors Associated with Self-Management

Table 5 shows the association of self-management with covariates. Univariate logistic regression showed associations of self-management with age, race, sex, marital status, social support, BMI, diabetes knowledge, and uncontrolled glucose (*p* ≤ 0.20). After controlling for potential confounders (sociodemographic, anthropometric, and physiological measurements, and medical status variables), the final hierarchical logistic regression showed significant associations of self-management of diabetes with sex [males: AOR = 0.55, 95% CI: 0.34–0.90], race [Coloured: AOR = 2.84, 95% CI: 1.69–4.77 and White: AOR = 3.84, 95% CI: 1.46–10.1], marital status [divorced: AOR = 3.41, 95% CI: 1.13–10.29], social support [average: AOR = 2.51, 95% CI: 1.05–6.00] and good: AOR = 4.49, 95% CI: 1.61–7.57], BMI [obesity: AOR = 0.31, 95% CI: 0.10–0.95], diabetes knowledge [average: AOR = 0.58, 95% CI: 0.33–0.10 and good: AOR = 1.86, 95% CI: 0.71–4.91], and uncontrolled glucose [AOR = 2.97, 95% CI: 1.47–5.98).

## 4. Discussion

Self-management among diabetic patients to achieve glycaemic control is one of the South African goals on Diabetes Implementation Strategy at the PHC level, developed in response to the African Diabetes Declaration and Strategy, yet the country still has the highest incidence of diabetes in the African continent [37,64]. We conducted a facility-based study to determine the diabetes self-management practices and associated factors among out-patients in Tshwane, South Africa. In evaluating all these factors jointly ( diet, exercise, blood sugar self-testing, medication, foot care, smoking, and alcohol consumption) and assigning a total self-management score, only 5% had good self-management, whilst 64% managed diabetes at an average standard, and 21% had poor self-management. Poor diabetic self-management accompanied by poor glycaemic control has been reported in LMICs and in SSA, including South Africa [20,24,34,39,40,41,42]. In South Africa, suboptimal management of diabetes has been reported, and only approximately 1 out of every 4 patients with T2DM are controlled [65,66,67]. As suggested in other studies [41,42], emphasis on compliance for all the various components of diabetes management through existing health education programmes at PHC is imperative. Amidst increasing cardiovascular risk factors [68], the burden of diabetes and poor management pose serious threats, considering the high rates of NCDs converging with HIV in the antiretroviral era [69,70,71] in South Africa and SSA. In addition, metabolic syndrome [72,73] accompanied by alarming rates of obesity [74,75,76] and the burden of hypertension [77,78,79,80] are also observed. As was reported in this study, hypertension (22%) was the common comorbidity and the prevalence of obesity (68%) was high. This prevalence of obesity confirms that patients struggled with exercise, receiving poor scores for this component.

Despite diabetes being a major public health concern, patients’ knowledge of various components of diabetes care, such as dietary practices, glucose testing, exercise, insulin use, complications identification, and screening, has been reported to be low in South Africa [42] and in countries such as Nepal [81]. Our study found an average level (55%) of diabetes knowledge among patients, which might impede glycaemic control and diabetes burden reduction. This corroborates the fact that poor knowledge is a common and persistent challenge in the management of diabetes. Furthermore, diabetes self-management is complex and requires several lifestyle modifications and engagement in certain behaviours to prevent complications and to improve health outcomes [82]. In South Africa, several PHC facilities offer diabetes education provided by nurses, doctors, and dietitians, and less frequently by professional diabetes educators [41]. Therefore, it is important that patients with diabetes be equipped with sufficient knowledge regarding diabetes self-management components through culturally sensitive education materials.

Most of the patients in this study were married and had attained secondary school education, but were from poor households with a monthly income between 83.11– 276.85 $. Socioeconomic inequalities are known to influence the prevalence of diabetes [83], and in South Africa, diabetes has been more commonly reported among the rich, though it is steadily rising among poor people [84]. In high-income countries, diabetes has been associated with low socioeconomic groups [85,86] and on the contrary, a high prevalence of diabetes is now reported among high socioeconomic status groups in LMICs [11,18,20]. Additionally, hypertension and high cholesterol have been reported as common comorbidity illnesses in this study, similar to other research [87]. The current study further showed poor diet, physical inactivity, overweight and obesity, heavy alcohol consumption, and heavy tobacco smoking among diabetes patients. This is consistent with the assertion that the dietary consumption of foods with excessive sugar, fat, and salt contribute remarkably to the high level of obesity in South Africa [41]. Lifestyle factors influencing diabetes inequalities in South Africa have been reported among rich people [88]. In addition, tobacco smoking and alcohol consumption reported in the current study contributed to self-management among diabetes patients. Smoking is well established as a risk factor for multiple diseases and has been associated with diabetes in South Africa [89] and other countries [90]. Similarly, alcohol consumption has been implicated to interfere with self-care behaviours and to affect important organs in the body [91]. The 33% proportion of alcohol consumption reported in this study is higher compared to the 1% proportion of heavy drinkers reported in a South African study, while current tobacco smoking is less in Gauteng province compared to Western Cape, Northern Cape, and Free State [39,92].

Then again, the importance of self-monitoring of blood glucose as an essential component of diabetes self-care and prevention of hypoglycaemia [93] can never be overemphasized, because of its guidance on medication and dosage adjustment, in addition to dietary intake and exercise regimes, and assisting the patient to be actively involved in achieving targeted glycaemic levels [39]. South Africa has recorded a high percentage of glucose self-testing among patients (92%) [88], which is attributed to most patients having a machine to self-test blood glucose [39]. However, in this study, diabetes patients performed poorly in self-glucose testing, despite half of the patients claiming to have glucometers at home. Possibly insufficient lancets or testing strips or patients lacking knowledge on the testing procedure might have hampered regular glucose self-testing beyond having glucometers. It is also worth noting that self-management activity on total medication score showed that two thirds of patients complied on average, which is almost comparable to local studies that estimated 67% to 70% medication adherence [39,94]. In other countries such as Ethiopia, poor self-glucose monitoring is estimated at 15.1% [40] among diabetes patients, and is estimated at 27% among Chinese Americans [95]. The variety of methods used to measure medication adherence affect the adherence outcome figures [96], and failure to adhere optimally to diabetes medication predisposes one to uncontrolled diabetes, which ultimately accelerates the development of diabetes complications such as retinopathy, nephropathy, and neuropathy [39].

Complications arising from diabetes are detrimental to the wellbeing of the affected individuals including medical and rehabilitative costs that come with hospitalisation, and financially demanding on the health system [34,35,36,87]. However, there is a scarcity of literature on educational management programmes in South Africa, such as foot-care management programmes, which are established clinical practice in other countries [87,97]. In South Africa, 69% of diabetes patients were reported to conduct foot care, while only 24% indicated that they were sufficiently aware that they had to conduct foot care [39]. Considering that in the current study, neuropathy, which usually comes with lack of sensation, is the most frequently reported complication in other countries such as Nigeria [98], foot care would be an important self-management practice. The current study showed that only 15% practiced the activity, while 12% inspected the inside of your shoes for any holes, thorns, small stones, or other abnormalities that may cause injury before putting them on daily. Therefore, this predisposes diabetes patients to ulcers, diabetes, food disease, and eventually amputation of lower limbs, suggesting a need for effective education on foot care.

In this study, the self-management of diabetes was significantly associated with sex, race, marital status, social support, BMI, diabetes knowledge, and uncontrolled glucose. Differences in diabetes-related factors may be due to variations in the study designs and the characteristics of the study populations [98]. For instance, a study in the United States of America, reported that men are less likely to engage in diabetes self-management education than women [99], as shown by the current findings. However, on the contrary, men have been reported to better self-manage diabetes than women in Nigeria [98]. Predominant barriers to self-care faced by men, such as lack of flexibility and schedule intensity within the workplace, have been implicated to hamper self-management [100]. While women demonstrate more positive outcome measures, including metabolic control, diet, and diabetes-related distress with increased self-confidence in living with diabetes and positive social support [101]. Possible reasons to the difference between males and females regarding glycaemic control has been attributed to differences in regulation of glucose homeostasis, treatment response, and psychological status [102]. The differences in diabetes self-management by sex may help inform sex-sensitive diabetes, care, counseling, and support, as suggested earlier [103]. Marital status was also associated with self-management in this study, similar to other reports in Ethiopia [104] and Brazil [105]. The benefits of improved health outcomes in relation to marital status have been previously suggested, and the hypothesis of a post-marriage effect, entailing a reduction in stress and the adoption of healthy behaviours [106,107], might play a role in our study population, and in this case, with divorced patients. Further, similar to the current study showing that patients from Coloured and White racial groups manage diabetes better than Black African patients, previous research has suggested that blacks in general have worse diabetes control than other racial groups [108]. Therefore, evidence is growing regarding race and ethnicity’s influence on individuals’ diabetes care. [108] This has been attributed to poor food choices, particularly foods high in carbohydrates and fat, hindering the management of diabetes [102].

Regarding other factors such as BMI, social support, diabetes knowledge, and uncontrolled glucose, the results of this study indicate that patients who are obese are less likely to participate in diabetic self-management practices. Obesity poses a significant risk for the development of insulin resistance, diabetes, and its subsequent complications [109]. Thus, diet and exercise are two of the greatest ways to reduce obesity, as one’s calorie intake is restricted, and energy expenditure is enhanced [110]. Furthermore, patients who receive social support from family/friends are more likely to totally self-manage, just as patients with average or good diabetes knowledge are more likely to self-manage diabetes. These sentiments are supported in South Africa [111], similar to the United States of America [112,113], indicating good social support among persons who had higher self-management scores, especially with reference to long-term care [114]. Therefore, healthcare providers should not neglect addressing the positive or negative role that family plays in a patient’s management of diabetes [108] through providing the right education to improve the ability of the family caregivers [114]. Furthermore, variations in socioeconomic status affect self-management either by the individual or family caregiver, with evidence that families from the higher socioeconomic groups show better care than those from low socioeconomic groups [115]. Then again, the literature documents that patients with good or average diabetes knowledge are more likely to carry out diabetes self-management activities compared to those with poor diabetes knowledge. This has been reported in other studies conducted in Africa, reporting that adequate diabetes knowledge translates to better self-management of diabetes [40,116]. Remarkably, this study found that patients having uncontrolled glucose levels are more likely to exhibit high self-management, which challenges logic. This should be investigated further in the future, considering that use of glycated hemoglobin (A1C, hemoglobin A1C, HbA1c) is the most appropriate measurement to reflect average levels of blood glucose over the previous two to three months [117].

In summary, our study reported more on the sociodemographic context, considering the person, place, and social risk factors implicated as obvious determinants of health [43,118]. This was in addition to observing the challenges to managing diabetes which revolve around diabetes knowledge, medication adherence, and other context barriers, as the literature documents [44,119]. Therefore, poor socioeconomic status, observed in this study, characterized by minimal household income and unemployment, in addition to race and gender, may have possibly posed as a barrier to optimal self-management of diabetes. In fact, poor socioeconomic status is associated with poor care [115], depriving patients full accessibility to health care resources due to financial and transportation issues. Furthermore, in most communities, self-management of any chronic disease is embedded within the cultural context, as well as patients’ experiences and behaviour, which are known to impede optimal care, resulting in poor health outcomes [120], and this might have been the case in the current study. Therefore, the self-management of diabetes appears to be shaped by the sociocultural context, and this calls for a better understanding of the contextual determinants to facilitate the development of culturally appropriate interventions to modify beliefs and support self-management in this population.

## 5. Limitations

First, the use of a cross sectional study design gives only inferences and limits any casual interpretations. This is coupled with the fact that this study was conducted within a short period of time (six months), although all the diabetes patients who came to the facilities at the time of data collection satisfied the inclusion criteria. Therefore, prospective cohorts are necessary to follow a larger number of patients with time and investigate causality. Second, although the facilities were randomly selected, they were not treated as a unit of analysis. The use of convenience sampling to select patients might have introduced bias, which we mitigated by obtaining a larger sample size. Third, we acknowledge a limitation regarding the classification of the duration of treatment into two groups, which does not account for the variability within each group. The duration of treatment was supposed to be collected as a value for proper classification and for the consideration of different experiences and outcomes. Fourth, the findings from this study are based on data collected from the four PHC facilities in Tshwane, and thus, may not be applicable to the whole of South Africa. In addition, the study was conducted among out-patients who had sought care in these facilities, and it might not be representative of the general population of diabetes patients. Fifth, data on diabetes knowledge were self-reported, which might have the disadvantages of recall and social desirability biases, leading to over or under estimation of some of the results. Furthermore, we did not establish whether the patients in the study had received health education on diabetes, which might have impacted their level of diabetes knowledge. Social desirability might also have been the case when obtaining information on self-management activities and practices. Sixth, considering that we did not measure diabetic control using glycated haemoglobin, all of the information that we received was self-reported by the patients. Future studies should assess glycated proteins in detail, primarily haemoglobin and serum proteins tests, which can quantify average glycaemia over weeks and months, thereby complementing day-to-day testing. Finally, future research, in particular, qualitative studies, or mixed method research, are necessary to respectively explore or understand the in-depth factors influencing diabetes self-care, considering facilitators and barriers within the context of diabetes patients. Nonetheless, our study provides insight into the self-management of diabetes among patients seeking chronic care in the selected PHC facilities in Tshwane, South Africa.

## 6. Conclusions

Diabetes knowledge among patients was average amidst prevalent uncontrolled glucose, obesity, hypertension, and high cholesterol comorbidities, as well as amidst neuropathy, eyesight problems, and amputation complications. A high proportion of diabetes patients achieved average self-management when diet, exercise, blood sugar self-testing, medication, foot care, smoking, and alcohol consumption were jointly evaluated. Factors associated with self-management were sex, race, marital status, BMI, social support, diabetes knowledge, and uncontrolled glucose. This calls for a better understanding of the contextual determinants to facilitate the development of culturally appropriate interventions to modify beliefs and support self-management in this population. Innovative approaches are perhaps needed to make diabetes education more effective. Face-to-face sessions delivered generally during clinic visits should be better tailored to the individual circumstances of people living with diabetes. Considerations should be given to the options of leveraging information technology to ensure the continuity of diabetes education beyond clinic visits. Additional effort is also needed to meet the self-care needs of all people living with diabetes.

## Figures and Tables

**Table 1:** Characteristics of out-patients and comparison by sex.

Variables	All	Males	Females	*p*-Value
	(*n* = 402)	(*n* = 192)	(*n* = 210)	
	*n* (%)	*n* (%)	*n* (%)	
**Age (years)**				
≤45	252 (63)	126 (66)	129 (61)	0.383
>45	147 (37)	66 (34)	81 (39	
**Place of residence**				
Urban	291 (72)	291 (72)	146 (76)	0.117
Peri-urban	111 (28)	111 (24)	46 (24)	
**Race**				
Black Africans	179 (45)	79 (41)	100 (48)	0.013 *
Whites	185 (40)	101 (53)	84 (40)	
Coloureds	33 (8)	12 (6)	21 (10)	
Indian/Asian	5 91))	0 (0)	5 (2)	
**Marital status**				
Single	161 (40)	82 (42)	79 (38)	0.005 *
Married	175 (44)	88 (46)	87 (41)	
Divorced	36 (9)	17 (9)	25 (12)	
Widowed	30 (7)	5 (3)	19 (9)	
**Level of education**				
No school/Primary	48 (12)	21 (11)	27 (13)	0.125
Secondary	136 (34)	76 (40)	60 (29)	
Completed grade 12.	198 (48)	85 (44)	107 (51)	
Post grade 12	26 (6)	10 (15)	16 (8)	
**Household income/month**				
<$83,06	20 (5)	8 (4)	12 (6)	0.228
$83,11–$276,85	214 (53)	104 (54)	110 (52)	
$276,91–$553,70	51 (13)	20 (10)	31 (15)	
$553,76–$830,55	65 (16)	38 (20)	27 (13)	
>830,55	52 (13)	22 (12)	30 (14)	
**Social support of family and friends**				
Poor	38 (9)	19 (10)	19 (9)	
Average	85 (21)	39 (20)	45 (21)	0.934
Good	280 (70)	134 (70)	146 (70)	
**Duration of diabetes diagnosis**				
<5	199 (49)	96 (50)	103 (49)	0.849
≥5	203 (51)	96 (50)	107 (51)	
**Have comorbid conditions**				
No	371 (92)	182 (95)	189 (90)	0.072
Yes	31 (8)	10 (5)	21 (10)	
**Comorbid conditions**				
High blood pressure (yes)	94 (24)	41 (21)	56 (27)	0.214
High cholesterol (yes)	86 (21)	44 (23)	42 (20)	476
Heart disease (yes)	16 (4)	10 (5)	6 (3)	0.228
Combination of the above (yes)	24 (6)	15 (81)	9 (5)	0.136
**Diabetes complications**				
Amputation (yes)	28 (7)	16 (8)	12 (6)	0.303
Kidney disease (yes)	21 (5)	12 (6)	9 (4)	0.377
Neuropathy (yes)	88 (22)	33 (17)	55 (26)	0.029 *
Eyesight problem (yes)	64 (16)	31 (16)	33 (16)	0.906
More than two of the above (yes)	25 (6)	12 (6)	13 (6)	0.89
**Ever been hospitalised**				
No	311 (77)	157 (82)	154 (73)	0.043 *
Yes	91 (23)	35 (18)	56 (27)	
**Blood glucose self-testing**				
No	198 (49)	93 (48)	105 (50)	0.754
Yes	204 (51)	99 (52)	105 (50)	
**Diabetes medication**				
Oral hypoglycaemics tablets (yes)	197 (49)	74 (39)	123 (59)	≤0.0001 *
Insulin (yes)	112 (28)	72 (38)	39 (19)	≤0.0001 *
Both above (yes)	85 (21)	40 (21)	45 (21)	0.884
Neither (yes)	8 (2)	5 (3)	3 (1)	0.399
**Diabetes knowledge**				
Good	61 (15)	28 (15)	33 (16)	0.165
Average	222 (55)	11 (60)	107 (51)	
Poor	119 (30)	49 (26)	70 (33)	

* Significance level at *p* < 0.05.

**Table 2:** Comparison of anthropometric and physiological measurements of patients by sex.

Variables	All	Males	Females	*p*-Value
	(*n* = 402)	(*n* = 192)	(*n* = 210)	
	*n* (%)	*n* (%)	*n* (%)	
**Glucose (mmol/L)**				0.012 *
controlled	313 (78)	160 (83)	153 (73)	
uncontrolled	89 (22)	36 (17)	57 (27)	
**Hypertension (mmHg)**				0.725
normal	286 (71)	135 (70)	151 (72)	
hypertension	116 (29)	57 (30)	59 (28)	
**BMI (Kg/m^2^)**				0.007 *
normal	29 (7)	15 (8)	14 (7)	
overweight	122 (30)	72 (38)	50 (24)	
obese	251 (63)	105 (55)	146 (70)	
**WC (cm)**				0.450
normal	27 (7)	11 (6)	16 (8)	
abdominal obesity	375 (93)	181 (94)	199 (92)	
**WHR**				0.386
normal	23 (6)	13 (8)	10 (5)	
abdominal obesity	379 (94)	179 (93)	200 (95)	
**WHtR**				0.338
normal	1 (1)	0 (0)	1 (1)	
abdominal obesity	401 (99)	192 (100)	209 (99)	

BMI stands for body mass index, WC stands for waist circumference, WHR stands for waist hip ratio, WHtR stands for waist to height ratio, F stands for female, and M stands for male, * *p* < 0.05.

**Table 3:** Self-management activities over the past seven days.

Self-Management Activities	Mean ± SD	Categories	*n* (%)
Diet On how many of the past seven (7) days did you eat five or more servings of fruit and/or vegetables? High fat foods On how many of the past seven (7) days did you eat high fat foods such as red meat of full-fat dairy products?	3.4 ± 2.0 3.4 ± 1.6	Poor Fair Good Poor Fair Good	190 (47) 166 (41) 46 (11) 197 (49) 174 (43) 31 (8)
Exercise On how many of the past seven (7) days did you participate in at least 30 min of continuous exercise? (Total minutes of continuous activity, including walking)	1.6 ± 2.1	Poor Fair Good	282 (70) 66 (16) 54 (13)
Blood glucose self-testing On how many of the past seven (7) days did you test your blood sugar?	1.1 ± 1.4	Poor Fair Good	373 (93) 21 (5) 8 (2)
Feet days On how many of the past seven (7) days did you check your feet for any wounds, skin changes or ingrown toenails? Shoe care On how many of the past seven (7) days did you inspect the inside of your shoes for any holes, thorns, small stones, or other abnormalities that may cause injury	3.0 ± 2.2 2.4 ± 2.2	Poor Fair Good Poor Fair Good	243 (60) 99 (25) 60 (15) 280 (70) 73 (18) 49 (12)
Medication On how many of the last seven (7) days did you take your diabetes medication as recommended?	5.8 ± 1.5	Poor Fair Good	38 (10) 93 (23) 271 (67)
Smoking If you are a smoker, on how many of the past seven (7) days did you smoke? How many cigarettes did you smoke on average every day? _______cigarettes I do not smoke	2.2 ± 1.5	Non Light Heavy	264 (66) 39 (10) 99 (25)
Alcohol consumption If you drink alcohol, on how many of the past seven (7) days did you drink alcohol? How many alcoholic beverages did you drink on average per day? _______drinks I do not drink alcohol	1.0 ± 1.6	Non Light Heavy	262 (65) 7 (2) 133 (33)

**Table 4:** Combined scores of self-management activities over the last eight weeks.

Combined Scores	Mean ± SD	Categories	*n* (%)	Range
Total diet score	9.77 ± 2.3	Poor	29 (7)	2–17
(Mean fruit/veg and inversion high fat)		Fair	333 (83)	
+ Sweets + Good diet =17		Good	40 (10)	
Total exercise score	6.99 ± 3.76	Poor	238 (59)	2–17
		Fair	126 (31)	
Exercise days + Avoid exercise + Physical activity = 17		Good	38 (10)	
Total medication score	9.89 ± 2.26	Poor	11 (3)	3–12
Medication days + Forgetting medication = 12		Fair	90 (22)	
		Good	301 (75)	
Total self-testing score	3.69 ± 2.20	Poor	289 (72)	1–12
Testing days + Measure = 12		Fair	99 (25)	
		Good	14 (3)	
Total self-management	41.5 ± 8.2	Poor	125 (31)	21–71
(Total diet score, total exercise score, total medication score, total self-testing score, foot care (Mean feet days + shoes days), smoking (1 if smoker, 0 if non-smoker), alcohol (1 if drinker, 0 if non-smoker), doctor, and self-care = 77				

**Table 5:** Association of self-management with patients’ characteristics.

Self-Management	OR (95%CI)	*p*-Value	AOR (95%CI)	*p*-Value
**Sex**				
Females	1		1	
Males	0.56 (0.37–0.86)	0.008 *	0.55 (0.34–0.90)	0.017 *
**Race**				
Black	1			
Coloured	1.58 (1.01–2.46)	0.045 *	2.84 (1.69–4. 77)	≤0.0001 *
White	1.82 (0.78–4.28)	0.166	3.84 (1.46–10.1)	0.006 *
Other	2.34 (0.25–21.35)	0.452	2.70 (0.25–29.5)	0.417
**Marital status**				
Single	1			
Married	1.19 (0.75–1.89)	0.451	1.39 (0.83–2.34)	0.216
Divorced	2.52 (0.92–6.96)	0.074	3.41 (1.13–10.29)	0.029 *
Widowed	0.79 (0.38–1.67)	0.542	0.86 (0.36–2.04)	0.731
**Social Support**				
Poor	1			
Average	2.86 (1.04–4.94)	0.041 *	2.51 (1.05–6.00)	0.038 *
Good	2.91 (1.46–5.80)	0.002 *	4.49 (1.61–7.57)	0.002 *
**BMI**				
Normal	1			
Overweight	0.64 (0.25–1.82)	0.402	0.74 (0.23–2.36)	0.617
Obesity	0.37 (0.14–1.01)	0.052	0.31 (0.10–0.95)	0.040 *
**Uncontrolled**				
**glucose**				
<7.0 mmol/l	1			
≥7.0 mmol/l	2.94 (1.59–5.45)	0.001 *	2.97 (1.47–5.98)	0.002 *
**Diabetes knowledge**				
Poor	1			
Average	0.66 (0.41–1.06)	0.087	0.58 (0.34–0.10)	0.049 *
Good	3.09 (1.28–7.45)	0.012 *	1.86 (0.71–4.91)	0.209

* Indicate *p <* 0.05.

## Data Availability

The dataset for the study group generated and analysed during the current study is available from the corresponding author upon reasonable request due to ethical restrictions.

## Supplementary Materials

The following supporting information can be downloaded at: https://www.mdpi.com/article/10.3390/ijerph20105887/s1, DIABETES SELF-MANAGEMENT QUESTIONNAIRE.
