# Supplementary material for: Self-Management of Diabetes and Associated Factors among Patients Seeking Chronic Care in Tshwane, South Africa: A Facility-Based Study
Source: Int J Environ Res Public Health. 2023 May 19;20(10):5887. doi: 10.3390/ijerph20105887 (PMC10218307; doi:10.3390/ijerph20105887)
Supplement: Supplementary file 1 [file ijerph-20-05887-s001.zip › ijerph-2282268-supplementary.pdf]

DIABETES SELF-MANAGEMENT QUESTIONNAIRE

PROJECT TITLE

SELF-MANAGEMENT PRACTICES AND ASSOCIATED FACTORS IN DIABETIC PATIENTS IN TSHWANE, SOUTH AFRICA

Greetings to participants and introduction of the study

"Thank you for agreeing to participate in this study. This study is about self-management practices and associated factors in diabetic patients in Tshwane, South Africa. The questionnaire is divided into four sections, namely, general and Socioeconomic questions, diabetes knowledge, self-management practices and anthropometry. We are starting with measuring your weight, height, waist, and hip circumferences, as well as anthropometry. Thereafter, we would request that you fill the questionnaire to best of your ability. For some questions you are welcomed to refer to your medical records. If there is a question you do not understand, please feel free to ask. I am here as a main researcher, together with the research assistants. Kindly feel free to request a questionnaire written in a language you prefer: English, Afrikaans or isiZulu".

Instructions:

1) You must be diagnosed with diabetes, above 18 years of age, and on treatment for more than 1 year.
2) Answer all questions. You are requested to be as honest as possible. There is no correct or incorrect answer.
3) Please fill in the answer or tick the appropriate box as requested.
4) Please do not hesitate to ask if you are uncertain of the meaning of a question.
5) Please do not write your name on the questionnaire.

Participant number: ________________ (For office use only)

**Section A: General and Socioeconomic Questions**			
**1) What is your current age?**		____________________ years	
**2) Please specify your race. Tick the appropriate box that applies to you.**			
Black Africans		Coloured	
Indian/Asian		White	
**3) Please specify your gender. Tick the appropriate box that applies to you.**			
Male		Female	
**4) What is your marital status? Please tick the box that applies to you**			
Single		Widowed	
Married			
Divorced			
**5) How would you describe your place of residence? Please tick the box most appropriate to you**			
Urban			
Peri-urban (i.e., Township)			
**6) How long ago were you diagnosed with diabetes? Please tick the box that applies to you**			
>1 year, but less than five (5) years ago		More than five (5) years ago	
**7) What level of social support do you receive from friends and family? Tick the appropriate box that applies to you.**			
Good		Average	
Poor			

**8) What is your household's total monthly net income? (Income before taxes). This can be earned via formal employment, informal employment, or a government grant.**		
Less than R1500	R1501 – R3000	R3001-R5000
R5001-R10 000	R10 001- R15 000	More than R15 001

**9) What is your highest level of education? Tick the appropriate box.**			
Did not attend school		Completed Grade 12	
Primary education		Tertiary education	
Secondary education			
**10) Do you suffer from any of the following medical conditions? Please tick the appropriate box if the condition applies to you.**			
High blood pressure		High cholesterol	
Heart disease		None	

**11) Do you have any other comorbidities/illnesses besides those stated above? If yes, please specify**			
Yes<br>Namely:		No	
**12) Do you have any of the following complications related to diabetes? Please tick the appropriate box if the condition applies to you**			
Amputation of toes/foot/leg		Kidney failure/ kidney disease	
Poor sensation in feet		Loss of eyesight	
None			
**13) Have you been hospitalized due to any complications related to diabetes?**			
Yes		No	
**14) Do you have access to a blood glucose machine to self-monitor your blood glucose levels at home?**			
Yes		No	
**15) Please tick what medication you are on to help manage your diabetes**			
Oral hypoglycaemic medication (tablets)		Insulin	
Both above		Neither	

Section B: Diabetes Knowledge

This section deals with your knowledge about diabetes. For each question, please circle the option (a, b, c, d) that you think is the most correct answer

16) For a person in good control, what effect does exercise have on blood glucose?

a) Lowers it
b) Raises it
c) Has no effect

17) A low blood glucose reaction may be caused by:

a) Heavy exercise
b) Infection
c) Overeating
d) Not taking your insulin

18) Which of the following is highest in carbohydrates?

a) Baked chicken
b) Cheese
c) Baked potato
d) Peanut butter

19) Eating food lower in fat decreases your risk for:

a) Nerve disease
b) Kidney disease
c) Heart disease
d) Eye disease

20) The best way to take care of your feet is to:

a) Look at and wash them each day
b) Massage them with alcohol each day
c) Soak them for 1 hour each day
d) Buy shoes larger size than usual

21) Numbness and tingling may be symptoms of:

a) Kidney disease
b) Nerve disease

c) Eye disease
d) Liver disease

22) Signs of ketoacidosis (DKA) include:

a) Shakiness
b) Sweating
c) Vomiting
d) Low blood glucose

23) A low blood glucose reaction may be caused by:

a) Too much insulin
b) Too little insulin
c) Too much food
d) Too little exercise

24) If you are beginning to have a low blood glucose reaction, you should:

a) Exercise
b) Lie down and rest
c) Drink some juice
d) Take rapid acting insulin

25) Which is the best method for home glucose testing?

a) Urine testing
b) Blood testing
c) Both are equally good

Section C: Self-management practices

26) On which of the following have you received self-management practice advice and education and have been instructed on to do, by your healthcare professional (for example, doctor or nurse). You may have multiple answers. Tick the appropriate box/boxes.

Diabetic diet		Foot care	
Exercise		Smoking	
Blood sugar testing		Alcohol consumption	
Diabetes medication use		None of the above	

The following few questions relates to your diabetes self-management activities over the past seven (7) days. For each question, please circle the appropriate number of days that applies to you.

Diet

27) On how many of the past SEVEN (7) DAYS did you eat five or more servings of fruit and/or vegetables?

0 1 2 3 4 5 6 7

28) On how many of the past SEVEN (7) DAYS did you eat high fat foods such as red meat of full-fat dairy products?

0 1 2 3 4 5 6 7

Exercise

29) On how many of the past SEVEN (7) DAYS did you participate in at least 30 minutes of continuous exercise? (Total minutes of continuous activity, including walking)

0 1 2 3 4 5 6 7

Blood sugar testing

30) On how many of the past SEVEN (7) DAYS did you test your blood sugar?

0 1 2 3 4 5 6 7

Foot care

31) On how many of the past SEVEN (7) DAYS did you check your feet for any wounds, skin changes or ingrown toenails?

0 1 2 3 4 5 6 7

32) On how many of the past SEVEN (7) DAYS did you inspect the inside of your shoes for any holes, thorns, small stones, or other abnormalities that may cause injury?

0 1 2 3 4 5 6 7

Medication

33) On how many of the last SEVEN (7) DAYS did you take your diabetes medication as recommended?

0 1 2 3 4 5 6 7

Smoking

34) If you are a smoker, on how many of the past SEVEN (7) DAYS did you smoke?

0 1 2 3 4 5 6 7

How many cigarettes did you smoke on average every day? _______cigarettes

I DO NOT SMOKE ☐

Alcohol consumption

35) If you drink alcohol, on how many of the past SEVEN (7) DAYS did you drink alcohol?

0 1 2 3 4 5 6 7

How many alcoholic beverages did you drink on average per day? _______drinks

I DO NOT DRINK ALCOHOL	☐

The following few questions contain statements that describe self-management activities related to diabetes. Thinking about your self-management over the last eight (8) weeks, please rate the extent to which each statement applies to you.

**Please tick to what extent the following self-management activities apply to you**	**Applies to me very much**	**Applies to me considerably**	**Cannot say specifically**	**Applies to me somewhat**	**Does not apply to me**
36) Occasionally I eat lots of sweets and other foods rich in carbohydrates					
36) I strictly follow dietary recommendations made by my doctor or relevant healthcare worker					
38) I check my blood sugars daily with care and attention					
39) I tend to forget to take my diabetes medication as recommended					
40) I avoid physical exercise, even though I know it would improve my diabetes					
41) I do regular exercise to achieve optimal blood sugar levels					
42) I keep my regular doctors' appointments recommended for my diabetes treatment					
43) My diabetes self-management is poor					

Section D: Anthropometric Measurements

The following measurements need to be taken by the researcher.

Body weight	kg	Waist circumference	cm
Body height	m	Hip circumference	cm

Blood glucose<br>(Last 3 measurements in file if available)<br>1<br>2<br>3	 <br><br>mmol/L<br>mmol/L<br>mmol/L	Blood pressure<br>(To be taken twice)<br>1SBP<br>2SBP<br><br>1DBP<br>2DBP	

----------------------------END--------------------------

Thank you for your participation in the study

Researcher's name ……………………………

Contact details…………………………………
